# Frontal axial pattern flap combined with hard palate mucosa transplant in the reconstruction of midfacial defects after the excision of huge basal cell carcinoma

**DOI:** 10.1186/s12957-018-1421-7

**Published:** 2018-06-29

**Authors:** Junliang Wu, Yong Qing, Ying Cen, Junjie Chen

**Affiliations:** Department of Plastic and Burn Surgery, West China School of Medicine, West China Hospital, Sichuan University, No.37, Guoxue Alley, Chengdu, 610041 Sichuan Province China

**Keywords:** Frontal axial pattern flap, Basal cell carcinoma, Midface reconstruction

## Abstract

**Background:**

Our article describes our experience with using a frontal axial pattern flap combined with hard palate mucosa transplant to reconstruct midfacial defects after the excision of huge basal cell carcinoma.

**Methods:**

We retrospectively reviewed four patients diagnosed with midface huge basal cell carcinoma through biopsy between 2014 and 2016. Both the eyelid and nose were involved in all the patients. All the patients underwent the studied surgical method and were followed up from 12 to 36 months.

**Results:**

All the patients preserved good eyelid function and relatively good esthetic satisfaction. No basal cell carcinoma recurred.

**Conclusions:**

This combined surgical procedure is a good method for reconstructing defects after the excision of huge basal cell carcinomas in the midface.

## Background

Basal cell carcinoma (BCC) is the most common non-melanoma skin cancer in the world. It primarily occurs in areas of the skin that are exposed to the sun, such as the face, neck, and trunk. There are many treatments, such as irradiation, cryotherapy, and topical application of a chemical such as 5-FU [1], but the most common radical approach is surgical excision [2]. Extended resection based on frozen section diagnosis during operation is unavoidable and leads to large defects in the face. The affected areas are mainly in sun-exposure sites. Midfacial areas are among the most commonly affected areas, and especially in the case of huge midface lesions, the eyelid and nose are often involved. The challenge in reconstructing the midface after ablative surgery is not only recovering functions but also preserving esthetics, especially for patients whose lesions involve the nose and eyelids. As we know, the eyelid is divided into two layers, that is the anterior skin-muscle and the posterior tarso-conjuctival lamella. When the two eyelid layers were defected, both layers need to be repaired in a way that preserves function and esthetics [3]. The hard palate mucosa provides an ideal option for the reconstruction of the eyelid inner layer because the donor site is in a concealed location and can heal itself [4–7]. Because the forehead area is non-hair bearing and relatively thin and has a color and texture similar to that of the midface [8], frontal axial pattern flaps such as the frontal branch flap of superficial temporal artery and the supratrochlear artery flap [9–11] are ideal choices for resurfacing huge midface defect. Therefore, frontal axial pattern flap combined with hard palate mucosa transplant might be a suitable method for reconstructing these defects.

In this retrospective study, we analyzed our experiences with midfacial reconstruction after the resection of huge BCC and concluded that forehead flaps combined with hard palate mucosa transplant are the most versatile reconstruction method in such cases, especially for older patients, because they are unable to tolerate the lengthy surgery required for a free flap transplant.

## Methods

From January 2012 to January 2014, four patients underwent surgical management of BCC with reconstruction of midfacial defects, including the nose and eyelids (three male patients and one female; age range, 65–82 years). All four patients were diagnosed with BCC by tissue biopsy and received frozen section diagnosis during surgery to identify the excision area. The follow-up period ranged from 12 to 36 months. The data of the four patients are shown in Table 1.

**Table 1:** Patients and profiles

Patient	Sex	Age (year)	Recurrence	Complication	Follow-up (months)
1	Male	82	No	Lower eyelid ectropion	18
2	Male	77	No	Flap color change	24
3	Female	65	No	Flap color change	36
4	Male	79	No	None	30

### Surgical procedure

**Fig. 1:**
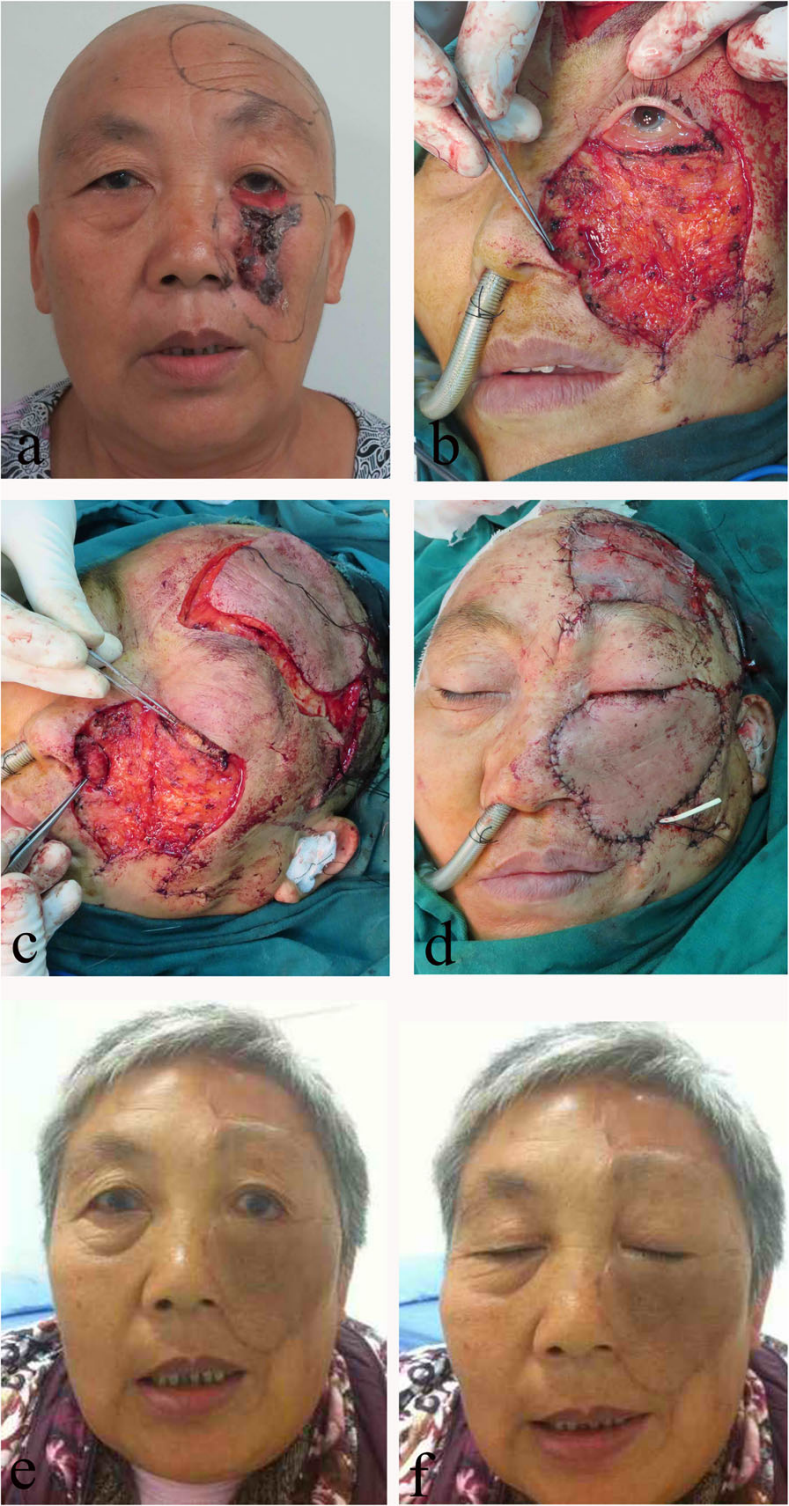
A 65-year-old woman with a huge left middle face BCC was treated by the present technique. **a** The frontal view before the operation. **b** The defect left after extensive resection, the left lower eyelid totally defect and left lower lateral nasal cartilage defect. **c** Hard palate(blue arrow) transplant to reconstruct inner layer of lower eyelid and auricular cartilage to reconstruct lower lateral nasal cartilage. **d** The frontal branch flap of superficial temporal artery was used to cover the middle face defect, the flap donor site received free skin graft. **e**, **f** 3 years after operation, no tumor recurred, the patient acquired good esthetic effect and relatively normal open and close eye function, but a little flap had color change

**Fig. 2:**
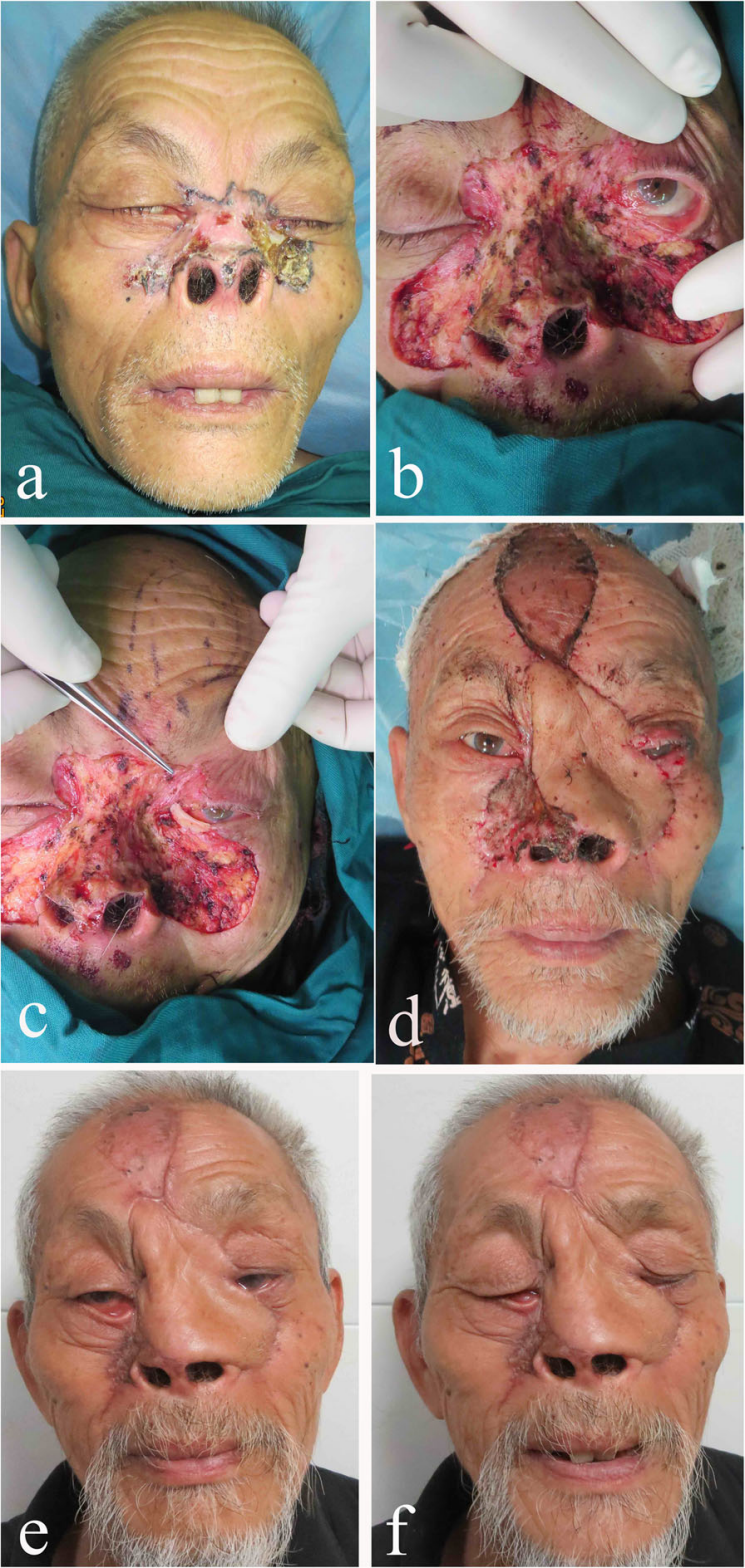
A 82-year-old man with a huge middle face BCC was treated by the present technique. **a** The frontal view before the operation. **b** The defect left after extensive resection, the left lower eyelid partial defect, and nasal bone exposure. **c** Hard palate transplant (blue arrow) to reconstruct inner layer of lower eyelid. **d** The supratrochlear artery flap was used to cover the middle face defect, the flap donor site, and the other site where the flap could not cover received free skin graft. **e**, **f** 1.5 years after operation, no tumor recurred, the patient acquired good esthetic effect and relatively normal open and close eye function (left side), but the right side had lower eyelid ectropion because of the free skin graft contracture

The four patients had huge BCC in the midfacial area (Figs. 1a and 2a). All procedures occurred under general anesthesia. First, the lesions were extensively resected according to frozen section pathology until no residual tumor cells were seen under microscope in the periphery and the basal aspect. After tumor removal, the four patients had partial or total full-thickness lower eyelid defects (Figs. 1b and 2b), and two had nasal bone exposure (Fig. 2b). Second, because of the lower eyelid defect after tumor removal, we excised a piece of hard palate mucosa tissue to reconstruct the inner layer of the lower eyelid (Figs. 1c and 2c). Then, we designed two different frontal axial pattern flaps—the supratrochlear artery flap or the frontal branch flap of the superficial temporal artery to cover the midfacial defect, and the flap donor sites received free skin grafts (Figs. 1d and 2d). If the defect was too large to cover using the flaps described above, we used the flaps to cover the exposed bone and the hard palate mucosa transplant area. The residual area then received free skin grafts (Fig. 2d).

## Results

All patients attained relatively normal lower eyelid function and no tumor recurrence during follow-up and found their reconstructed midface areas esthetically satisfactory (Figs. 1e, f and 2e, f). One patient had lower eyelid ectropion because of contralateral free skin graft contracture (Fig. 2e). Two patients experienced changes in the flap color (Fig. 1e).

## Discussion

BCC is the most common cutaneous cancer in the midface area and is characterized by local spreading and an exceptionally rare tendency to metastasize. Radical excision has a significant advantage compared with other treatments because surgical excision ensures the highest chance of cure. Therefore, the main treatment for BCC is surgery, and the safe margins of the excised tumor excised are mainly decided by intraoperative frozen section diagnosis. Excision defects of the middle face may be covered with skin grafts, regional or local flaps, or even free flaps.

A local skin flap is a geometric segment of tissue contiguous with a skin defect that is advanced, rotated, or transposed to resurface the defect. With topographical consideration, local flap is the preferred choice for the repair of small defects. However, the reconstructive modality of choice will depend on location, size, and depth of the surgical defect. For the patients in our group who had huge BCC in the midface, the lesions often affected the cheek, eyelids, and nose; after radical excision, the patients had a different whole-layer eyelid defect and nose defect, and some had nose bone exposure or a large cheek defect. Obviously, local flaps could not resurface these defects. Reconstructing these defects with adequate shape, color, and texture presents a significant problem for the plastic surgeon. Furthermore, most of the patients were elderly. Elderly people usually cannot tolerate a lengthy operation such as free flap transplant, and their vessels are not appropriate for free flap transplant because of angiosclerosis of the small vessels.

The patients in our study required periocular and eyelid reconstruction. The reconstruction should ensure normal eyelid function and focus on eye protection [11]. The eyelid is divided into two layers, the anterior skin-muscle and the posterior tarso-conjuctival lamella. Both layers need to be repaired in a way that preserves function and esthetics [3]. When the eyelid is resected, the hard palate mucosa provides an ideal option for the reconstruction of the inner layer because the donor site is in a concealed location and can heal itself [4]. Because the forehead area is non-hair bearing and relatively thin and has a color and texture similar to that of the midface [8], frontal axial pattern flaps are ideal choices for resurfacing midface defect. Therefore, frontal axial pattern flap combined with hard palate mucosa transplant is a suitable method for reconstructing these defects. The hard palate mucosa can be used to reconstruct the inner layer of the lower eyelid, and the tarsal plate no longer needs to be considered. The flaps can cover the free hard palate mucosa transplant, the exposed nose bone, and free auricular cartilage transplant used to reconstruct the nasal alar.

Frontal axial pattern flaps include two different flaps. One is the frontal branch flap of the superior temporal artery and the other is supratrochlear artery flap. Most huge midfacial defects can be reconstructed with those two flaps. In the central part of midface, we usually select supratrochlear artery flaps; in the cheek part, we can select superior temporal artery frontal branch flaps. After long-term follow-up, the reconstructed eyelid had good function and esthetics. If the defect is too big to resurface with a flap alone, a free skin graft should be used to cover the area where a skin graft cannot be used, such as the free hard palate mucosa transplant, free auricle cartilage graft, and bone exposure area. Other areas can be resurfaced with a free skin graft. Through this method, we achieved acceptable esthetic and functional results.

## Conclusions

While there are many approaches to reconstructing midfacial defects, none of them are fully satisfactory. For older patients who need eyelid reconstruction and coverage of the exposed nasal bone, frontal axial pattern flaps combined with hard palate mucosa transplant is an ideal method for reconstructing the defect. In our study, after long-term follow-up, the patients had good eyelid function and relatively good esthetic contours.
